# Human Papillomavirus E6 and E7: The Cervical Cancer Hallmarks and Targets for Therapy

**DOI:** 10.3389/fmicb.2019.03116

**Published:** 2020-01-21

**Authors:** Asmita Pal, Rita Kundu

**Affiliations:** Cell Biology Laboratory, Department of Botany, Centre of Advanced Studies, University of Calcutta, Kolkata, India

**Keywords:** human papillomavirus, oncoproteins E6 and E7, cervical cancer, cancer hallmarks, E6 targeted gene therapy, phytotherapy, immunotherapy

## Abstract

Human papillomavirus (HPV)-induced cervical cancer is a major health issue among women from the poorly/under-developed sectors of the world. It accounts for a high-mortality rate because of its late diagnosis and poor prognosis. Initial establishment and subsequent progression of this form of cancer are completely dependent on two major oncogenes E6 and E7, which are expressed constitutively leading to tumorigenesis. Thus, manipulation of these genes represents the most successful form of cervical cancer therapy. In the present article, information on structural, functional, and clinical dimensions of E6 and E7 activity has been reviewed. The genome organization and protein structure of E6 and E7 have been discussed followed by their mechanism to establish the six major cancer hallmarks in cervical tissues for tumor propagation. The later section of this review article deals with the different modes of therapeutics, which functions by deregulating E6 and E7 activity. Since E6 and E7 are the biomarkers of a cervical cancer cell and are the ones driving the cancer progression, therapeutic approaches targeting E6 and E7 have been proved to be highly efficient in terms of focused removal of abnormally propagating malignant cells. Therapeutics including different forms of vaccines to advanced genome editing techniques, which suppress E6 and E7 activity, have been found to successfully bring down the population of cervical cancer cells infected with HPV. T-cell mediated immunotherapy is another upcoming successful form of treatment to eradicate HPV-infected tumorigenic cells. Additionally, therapeutics using natural compounds from plants or other natural repositories, i.e., phytotherapeutic approaches have also been reviewed here, which prove their anticancer potential through E6 and E7 inhibitory effects. Thus, E6 and E7 repression through any of these methods is a significant approach toward cervical cancer therapy, described in details in this review along with an insight into the signaling pathways and molecular mechanistic of E6 and E7 action.

## Introduction

Cervical cancer is continuing to rise as a global concern with around 570,000 cases diagnosed and 311,000 deaths registered in the year 2018 (Bray et al., 2018). This form of cancer although has started to show a decline in the developed countries is still the reason behind the maximum number of cancer deaths among women in around 43 less developed countries (Torre et al., 2017). Around 85% of all the cervical cancer deaths hail from these low income countries, which are 18 times higher than that of the developed countries (LaVigne et al., 2017). This incongruity in the mortality rate between the developed and the developing/underdeveloped countries is due to the lack of proper awareness, screening programs, inaccessibility to proper diagnosis, and efficient treatment procedures along with an increased exposure to the risk factors leading to cervical cancer. Besides the major risk factor-human papillomaviral (HPV) infection, poor hygiene conditions, smoking, oral contraceptives usage, exposure to diethylstilbestrol (DES), and genetic predisposition are particularly common among women from low socio-economic background (American Cancer Society, 2018). Such a clinical scenario definitely calls in for an inexpensive affordable targeted therapeutic approach. Targeted therapeutic approach targets the major factor responsible for cervical cancer, i.e., HPV infection. Although HPV infection cannot singly induce cervical carcinogenesis, it is a primary requisite for most of the cervical cancer cases. This review discusses in detail about the human papillomavirus genome and its major oncogenes E6 and E7, i.e., the builders of six hallmarks of cancer along with a deeper insight into the clinical implications of E6 and E7 disruption through modern day genome editing techniques or phytotherapeutic methods. Thus, this article tries to encompass all the structural features and the biological functions of E6 and E7 oncogenes along with its role as a therapeutic target, helping to gather a comprehensive knowledge about E6 and E7.

## Human Papillomavirus and Cervical Cancer

Human papillomaviruses from the family Papillomaviridae belong to a category of small non-enveloped circular double-stranded DNA viruses, measuring 50–55 nm in diameter (Doorbar et al., 2016). The category includes 300 different genotypes, among which 200 of them are known to be detrimental to humankind (Serrano et al., 2017). From nucleotide sequence comparison of the L1 ORFs of 118 papillomavirus types, papillomaviruses have been classified by the team of de Villiers et al. (2004) into genera, species, types, and subtypes. Including the modifications proposed by Bernard et al. (2010), papillomaviruses from human have been grouped into five genera: alpha (65 types including HPV16, 18, 31, 33, etc.), beta (53 types including HPV5, 9, 49, etc.), gamma (98 types including HPV4, 48, 50, etc.), mu (3 types including HPV1, HPV63, and HPV 204), and nu (HPV41; www.hpvcenter.se as accessed on December 19, 2019). Among them, alpha-papillomaviruses are the most commonly focused group of papillomaviruses since they are known to be responsible for 5% of the cancer occurrences worldwide (Egawa and Doorbar, 2017). They are further subdivided into cutaneous or mucosal types based on their ability to infect the epithelial skin cells or inner tissue lining, respectively, and either low-risk types or high-risk types depending on their association with cervical cancer or precancerous lesions. The high-risk HPVs include HPV16, 18, 31, 33, 35, 39, 45, 51, 52, 56, 58, and 59 of mucosal alpha type genus, classified as Group 1 carcinogens; while HPV68 is considered as a probable human carcinogen under group 2A with limited human evidences. On the other hand, HPV26, 30, 34, 53, 66, 67, 69, 70, 73, 82, 85, and 97 are considered as possible group 2B carcinogen by International Agency for Research on Cancer (IARC) with limited epidemiological reports (Bouvard et al., 2009). Among the high-risk types, HPV 16 is undoubtedly the most frequently detected genotype in 60.5% of cervical cancer cases, followed by HPV18 (Serrano et al., 2017).

Fortunately, HPV-infected cancers have become preventable by the discovery of prophylactic vaccines. There are three such vaccines accessible in the pharmacy, which include Cervarix (GlaxoSmithKline), Gardasil (Merck Inc.), and Gardasil 9 (Merck Inc.), which can target 2, 4, or 9 different HPV types, respectively. At the same time, the mortality chart for cervical cancer, however, does not reflect the clinical success of such vaccination process. The plausible explanation is the unavailability of these vaccines in the underdeveloped countries, which are the major ones building up the mortality percentage. Moreover, the women from the financially challenged section of the population are not exposed to enough screening and diagnostic procedures, owing to many socio-cultural and financial burdens. Since prophylactic vaccines can work only in the early phases, they cannot prevent the development of cervical cancer in such deprived category of population because of their late diagnosis. The scientific community is thus focusing on the development of therapies to combat cervical cancer, which have already established themselves in the women body. This can be facilitated through targeting of the major oncogenes of HPV-driven carcinogenesis, i.e., E6 and E7. E6 and E7 inhibition can help suppress cervical cancer development even in the advanced stages.

## Human Papillomavirus Genome and Its Integration Into the Host Cellular Genome

**Figure 1:**
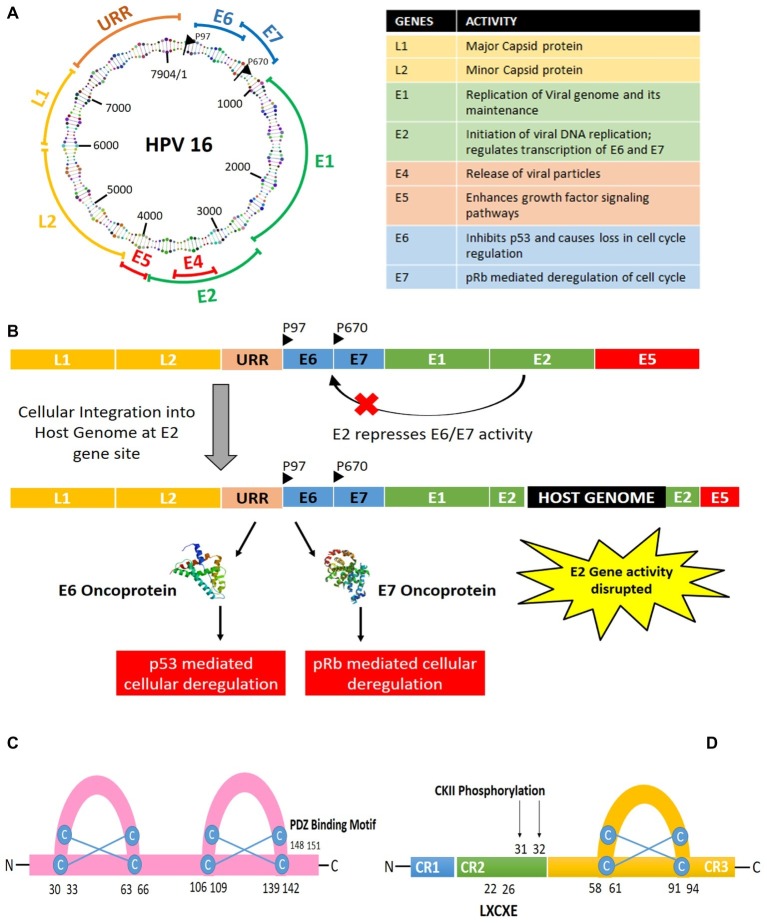
**(A)** Structure and organization of HPV16 genome. **(B)** Integration of HPV genome into the host genome *via* disruption of the E2 gene leading to the expression of the oncogenes E6 and E7. **(C)** Structure of E6 oncoprotein. **(D)** Structure of E7 oncoprotein.

The HPV genome structure and the oncogenes have been discussed in this section with reference to high-risk HPV16 type (Figure 1). The HPV16 genome is a 7.9 kb long nucleotide belt, segmented into three sections: the early gene-coding region (E), the late gene-coding region (L), and the long control region (LCR), also called as non-coding region (NCCR) or upstream regulatory region (URR). These gene segments are divided by two polyadenylation (pA) sites: early pA (A_E_) and late pA (A_L_). The 5′ end begins with the early gene coding region, which has six open reading frames, named as E1, E2, E4, E5, E6, and E7. E1 and E2 are known to regulate the replication of the viral genome and transcription of early proteins, while E5–E7 are the ones inducing oncogenesis. E5 is known to help in keratinocyte differentiation and immune evasion during the later stages, while E6 and E7 take in charge of several cellular checkpoints to establish the cancer hallmarks. Hence, this review focuses on the significance of E6 and E7 oncoproteins only, termed as “oncoplayers” in this review. The late gene coding section has two parts: L1 and L2. L1 codes for a major viral capsid protein, while L2 codes for a minor viral capsid structure. Although the 850 bp stretch of LCR does not contain any protein coding sequence, it contains the origin of replication and numerous transcription factor binding sites for RNA polymerase II facilitated transcription.

HPV infection begins in the basal layer of the stratified squamous epithelium, wherein initially E1 and E2 take charge of the viral DNA replication at a low copy number. Later, when the basal cells differentiate to form the epithelial suprabasal layer, viral genome replication switches into high copy number mode. Then, the virions get released upon epithelia desquamation, causing infection in the neighboring cells. HPV genome can either get integrated with the host genome or stay in an episomal form, with 83% of the HPV-positive cervical cancer cases showing evidences of HPV genome integration into the host cell (Burk et al., 2017). In case of a viral genome integration with the host genome, it frequently leads to the disruption of E2 gene site. The E2 gene is responsible for repressing E6 and E7, thus causing E6 and E7 to get activated upon viral genome integration into the host genome. Throughout the course of infection, E6 and E7 activity are responsible for the multiplication of the viral genome with the help of the cellular machinery, as revealed by several interactome analyses (Neveu et al., 2012; White et al., 2012a,b). They can trick the cells to become oncogenic in the process of viral replication. Hence, HPV-mediated tumor development can be defined as a collateral damage of the viral infection.

## Human Papillomavirus E6 and E7 – the Oncoplayers

HPV E6 and E7 viral oncoproteins play the pivotal role in driving the cells toward oncogenesis. In their process of replicating the viral genome, they can induce all the hallmarks of a cancer cell, i.e., uncontrolled cellular proliferation, angiogenesis, invasion, metastasis, and unrestricted telomerase activity along with the evasion of apoptosis and growth suppressors’ activity. Several *in vitro* and xenograft studies have also shown cancer cells to senesce or undergo apoptosis in the absence of E6 and E7 activity (Yamato et al., 2008; Jabbar et al., 2009), thus proving the absolute requirement of E6 and E7 for persistence of HPV-mediated cancer.

Both E6 and E7 are transcribed polycistronically from a single promoter located at the 3′ end of the upstream regulatory region (URR). E6/E7 transcription is under the regulation of several transcription factors such as AP1 and SP1, which functions by binding to the URR region.

E7 was the first oncogene to be discovered, among all the HPV oncogenes. It is a relatively small phosphoprotein of about 100 amino acids, with three conserved regions 1/2/3 (CR1/2/3). A small portion of CR1 and nearly entire CR2 from the amino terminal holds sequence similarity with adenovirus (Ad) E1A proteins and large T antigen of SV40 (Phelps et al., 1988). The CR2 domain is composed of poorly conserved sequence followed by the CR3 region. The CR3 region at the carboxyl terminal end is conserved and encodes a zinc finger domain containing two CXXC motifs separated by 29 amino acid residues (Barbosa et al., 1990; McIntyre et al., 1993). It is responsible for the zinc-dependent dimerization and for mediating E7 interaction with cellular proteins responsible for cell cycle regulation and apoptosis (p21 and pRb; Ohlenschlager et al., 2006).

On the other hand, E6 is relatively larger protein with 150–160 amino acids coding an 18 kDa protein (Zanier et al., 2012). It is arranged into two zinc finger binding domains by four Cys-X-X-Cys motifs, which have been found to be responsible for the oncogenicity of the protein (Nomine et al., 2006). The carboxy terminal domain contains a PDZ-binding motif responsible for interacting with several cellular proteins (Thomas et al., 2002). Out of all the types of cellular interactions the oncoproteins undergo, the most significant interaction is the one where E6 can degrade p53 and in case of E7 is the inhibition of pRb protein.

Besides E6 and E7, E5 also plays a vital role in the process of oncogenesis. E5 is an 83 amino acid hydrophobic membrane-associated protein associated with endoplasmic reticulum. Initially, E5 from BPV was identified as an oncogene; later, HPV16 E5 was also proved to be oncogenic, which can induce carcinogenic transformation along with E6. It is known to induce aberrant cellular proliferation through ligand-mediated activation of EGFR, inhibit apoptosis through degradation of Fas receptors and prevention of formation of death domain, and help the carcinogenic cells evade immune trap to progress toward malignancy (reviewed by Venuti et al., 2011).

## Manipulating the Cancer Hallmarks

E6 and E7 can drive a cell toward malignancy by contributing to the six major hallmarks of cancer, through several molecular pathways. Detailed insights into the pathways targeted by the oncogenes E6 and E7 to induce each of the phenotypic hallmark of cancer have been described in this section.

### Evasion of Growth Suppressors

**Figure 2:**
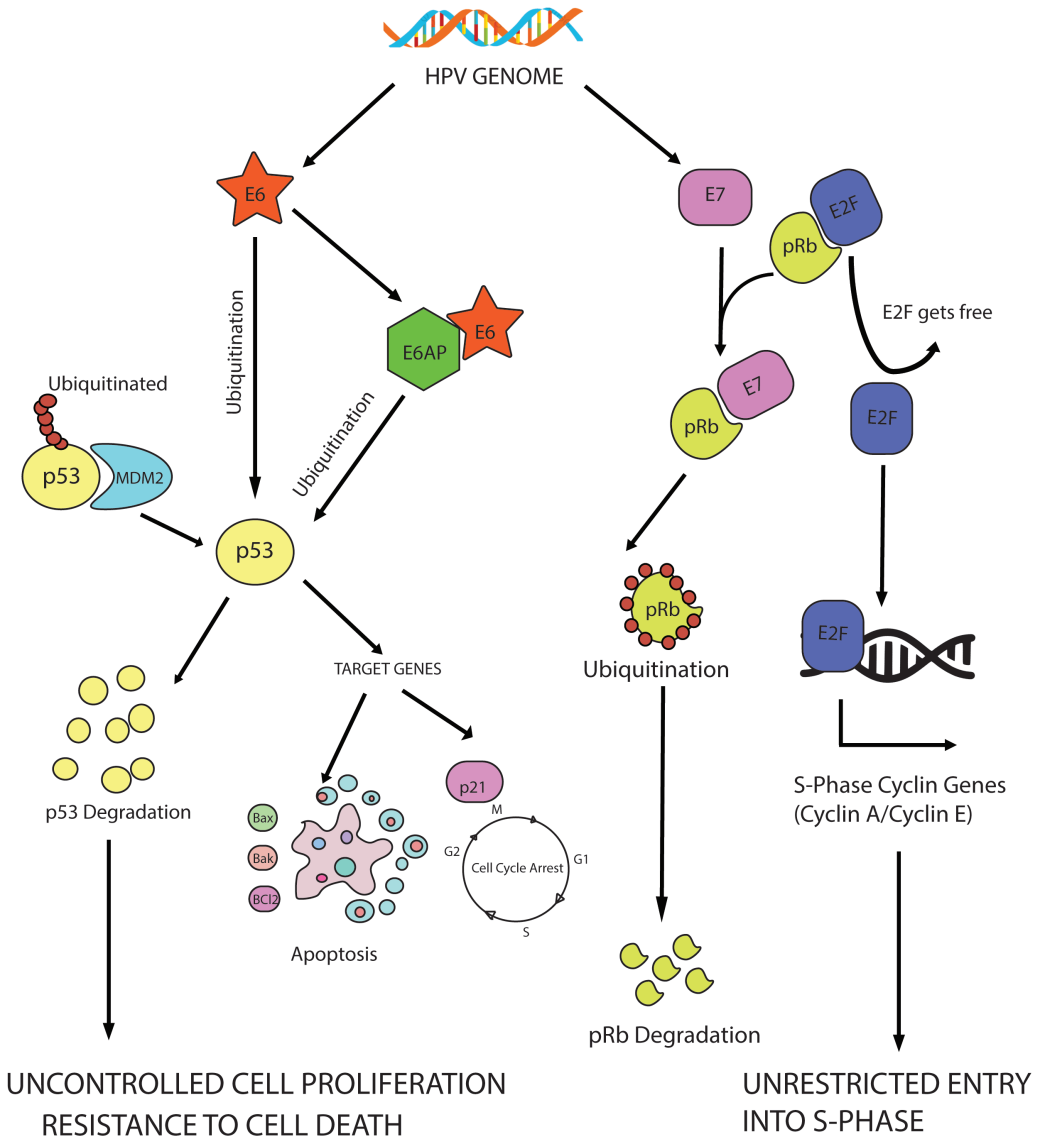
E6 mediated p53 manipulation and E7 mediated inhibition of pRb protein leading to sustained cell proliferation and resistance to apoptotic barrier.

Both E6 and E7 contribute to achieve uncontrolled proliferation through deregulation of growth suppressors. E6 targets an important growth suppressor, p53, while pRb is one of the major targets of E7 among few others. It has been diagrammatically illustrated in Figure 2.

E6-mediated inhibition of p53 allows several cellular changes to turn a cell oncogenic, one of them being induction of uncontrolled cell proliferation by evading the cellular checkpoints. The 53 kD molecular weight protein, p53, is the most well-characterized tumor suppressor protein till date and is often called as the “guardian of the genome” since it decides the fate of a cell during stressed conditions. When the cell experiences stress in the form of oxidative damage or other forms, it acts a transcription factor to transcribe the genes needed for either cell cycle arrest or apoptosis (Pflaum et al., 2014). On the other hand, murine double minute 2 (MDM2) homolog, an E3 ubiquitin ligase, helps to maintain it at a basal level in a healthy cell. Thus, p53 perturbation by E6 is significant to ensure continuous cellular proliferation.

E6 has been found to degrade p53 through ubiquitination with the help of E6AP (E6-associated protein also known as UBE3A; Scheffner et al., 1990, 1993). HPV E6 can bind to the LxxLL consensus sequence in the conserved domain of E6AP to form a heterotrimeric complex of E6/E6AP/p53, ultimately leading to the degradation of p53. This forces the cells through uncontrolled cellular division, evading the preventive checkpoints. Several *in vivo* experiments showed that interaction with E6AP is an absolute necessity to develop tumorigenecity in several tumor forms (Nguyen et al., 2002; Kelley et al., 2005).

Similarly, E7-mediated inhibition of retinoblastoma protein (pRb) is also a significant step toward achieving unrestrained cell proliferation. pRb–E2F interaction is a mandatory checkpoint for the cells to travel through G1-S phase transition. When the cells are not prepared to enter the S-phase, pRb protein remains bound to the E2F family of transcription factors to prevent them from transcribing the genes required in S phase. In HPV infected cells, E7 targets pRb for ubiquitination, leading to the release of E2F transcription factors, which transcribe cyclin E, cyclin A and p16^INK4A^, an inhibitor of CDK4/6, forcing the cells through premature S-phase entry (Boyer et al., 1996). CDK inhibitor p16^INK4A^ (tumor suppressor protein) is an important target of HPV E7 to regulate the cell cycle. HPV E7 triggers the expression of p16^INK4A^ not only through pRb disintegration but also by epigenetic derepression through KDM6B (H3K27-specific demethylase 6B; McLaughlin-Drubin et al., 2013).

HPV-E7 is also known to interact with DREAM (dimerization partner, RB-like, E2F4, and MuyB) complex and found to act downstream of p53 pathway (Rashid et al., 2011, 2015). DREAM complex helps to suppress the activity of cell cycle-related genes when not required (Sadasivam and DeCaprio, 2013). The LxCxE motif of HPV E7 can bind to the p130 of the DREAM complex to induce proteasomal degradation of the DREAM complex (Zhang et al., 2006). Hence, this E7-mediated disruption of DREAM complex is indispensable for cell cycle progression in cervical cancer (Rashid et al., 2011).

HPV-infected cells show co-expression of E6 and E7, which establishes the perfect environment for sustained proliferative signaling. The anomalous growth stimulus created by E7-mediated pRb disintegration could be stabilized by p53, which also gets hampered by E6, leading to evasion of all anti-tumorigenic checkpoints to drive the cells through cell division uncontrollably.

HPV E6 and E7 target another vital oncogene, c-myc, which has been claimed as a marker protein for several cancer forms, including cervical cancer. When disrupted by E6/E7, it has been found to disrupt cell proliferation, apoptosis, and cellular transformation. Peter et al. (2006) demonstrated the most noteworthy fact that the HPV genome integration takes place within the MYC locus (chromosome band 8q24), through FISH (fluorescence *in situ* hybridization) experiments. This is the reason c-myc expression is often altered in HPV infected cervical cancer cells. Deregulation of c-myc leads to disruption of Cdks, cyclins, and E2F transcription factors, as myc is capable of inducing cyclin/Cdk complexes with the help of Cdk activating kinase (CAK) and Cdc25 phosphatases. Myc is further found to reverse the Cdk inhibiting activity of p21 and p27 (Bretones et al., 2015). Besides this, both E6 and E7 can interact with c-myc to cause hTERT promoter activation, thus contributing to immortality of the cancer cells (Veldman et al., 2003; Liu et al., 2007).

In order to ensure continued cell proliferation, the HPV-infected cells need to bypass the mitotic spindle checkpoint too, which is also carried out by E6 and E7 together (Thomas and Laimins, 1998). E6 relies on p53-dependent pathway, while E7 evades the spindle checkpoint in a p53-independent manner, taking help of pRb. This was proved by Khan and Wahl (1998), wherein he showed that Rb deficient cells remain arrested at the spindle assembly checkpoint.

As a result of the alteration of several cell cycle regulators, the Cyclin-CDK complexes are the major players of cell cycle that gets transformed dramatically in HPV-infected cervical cancer cells. Cyclin D1-CDK4 and Cyclin D1-CDK2 associations get reduced in cells expressing E6 and gets completely abolished in cells expressing both E6 and E7. This has been linked to the reduced levels of p21 and activated levels of p16 (Xiong et al., 1996).

### Resisting Cell Death

When a cell is challenged with any unrepairable form of cellular damage through extrinsic or intrinsic factors, cells opt to die either in a programmed fashion (i.e. apoptosis) or through non-programmed way (i.e., autophagy or necrosis, etc.). This is a part of natural protective measure exerted by the body’s immune system to eliminate the cancer cells. Hence, in order to establish malignancy, HPV-infected cells rely on E6 along with E7 in order to escape the apoptotic protection.

At the molecular level, apoptosis is an act between two groups of proteins: pro-apoptotic and anti-apoptotic proteins, which function through two interconnected pathways: extrinsic and intrinsic pathway of apoptosis. The HPV oncoproteins disrupt or modulate these pathways to help the cancer cell escape the innate immune protection. E6 has been found to block apoptosis through both p53-dependent (Garnett and Duerksen-Hughes, 2006) and p53-independent manner. E6 of not only high risk HPVs but also low risk or cutaneous HPVs can directly interact with BAK leading to the degradation of BAK *in vivo* (Thomas and Banks, 1999). Moreover, BAK-mediated intrinsic mode of apoptosis can also be blocked by E6 through p53-E6AP interaction (Garnett and Duerksen-Hughes, 2006). A study in 1999 showed that when E6 is silenced, p53 levels increase resulting in activation of PUMA promoter, which causes Bax to drive the cell toward apoptosis through loss in mitochondrial membrane potential (Thomas and Banks, 1999). E6 is also noted to function independent of p53 as it can also hinder TNF-mediated extrinsic mode of apoptosis through the PDZ domain of E6, which can bind to TNFR1 and prevent TRADD from interacting with it (Filippova et al., 2002). E6 can interfere with the programmed pathway of apoptosis through several other targets such as FADD and caspase-8, which are directly targeted for ubiquitination (Yuan et al., 2012). Another striking activity of E6 was activation of the survivin promoter, which can also prevent apoptosis from taking place (Borbély et al., 2006). HPV E7 was found to have no such effect; instead, it showed minimal pro-apoptotic activity, which was nullified by the strongly anti-apoptotic consequences of E6 activity (Aguilar-Lemarroy et al., 2002). In addition to the apoptotic modulation, a noteworthy observation was made by Shankar et al. (2017), wherein TALEN-based editing of HPV E7 was found to induce necrosis in cervical cancer cells, which has been proved to hold better therapeutic response.

### Sustained Proliferative Signaling

A normal cell enters and progresses through cell cycle and divisional phases in response to the several growth promoting signals, which are produced and released in a controlled fashion. These signals are sensed by the receptor kinases present on the cell surface and transmitted inside the cells through several branched pathways. These pathways are the means to regulate the cellular dynamics related to survival or energy metabolism. In case of a cancer cell, these signaling pathways are deregulated through several means, which lead to sustenance of unrestricted proliferation. One such important signaling pathway helping in cell survival and proliferation is mediated by the oncogenic Ras. Mutated version of Ras helps to drive the cells to tumor progression through downstream effector pathways, like PI3K (phosphor-inositol-3kinase)-PKB (protein kinase B)/Akt and MAPK (MAP kinase) pathways. E6 has been found to activate the MAPK pathway through immunohistochemical analysis of organotypic raft cultures (Chakrabarti et al., 2004). Besides this, E6 and E7 have been found to have a deep implication on mTOR pathway to regulate cell proliferation too. HPV16 E7 expressing cells were found to undergo autophagy even in nutrient rich condition (Zhou and Munger, 2009). When nutrients were limited, E7 expressing cells unlike the normal cells would still continue to proliferate and ultimately lead to caspase-independent cell death, a process termed as “trophic sentinel response” (Eichten et al., 2004). But in the presence of E6, this can be avoided, as E6 is known to activate mTORC1 signaling to increase protein synthesis even in the absence of growth factors (Zhou et al., 2009; Spangle and Munger, 2010). Even in the absence of sufficient nutrient signals, E6 can activate mTORC1 through the upstream kinases PDK1 and mTORC2. PDK1 is located downstream of PI3K signaling pathway and is activated through several membrane associated receptors, such as ERBB, INSR (insulin receptor), IGFR (insulin-like growth factor receptor), and RPTKs (receptor protein tyrosine kinases). These receptors then interact with multiple signaling adaptor proteins such as GRB2 (growth factor receptor-bound protein 2) and SHC (Src homology 2 domain) to trigger the downstream cascade of AKT and mTORC1. A report by Spangle and Munger (2013) showed E6 expressing cells to hyperactivate and increase the internalization of phosphorylated RPTKs, even in the absence of nutrient factors. This leads to increase in mTORC1-dependent cellular growth and proliferation even in the absence of growth factors, helping in the tumor progression successfully.

### Enabling Replicative Immortality

With every new round of replication, the telomeres shorten in length as a part of cellular aging. Hence, in case of a cancer cell, cells need to prevent the telomeres from shortening in order to sustain the tumor progression. Telomerase is the enzyme, which is responsible for the chromosomal end replication; thus, it is overexpressed in case of a cancer cell and inactive in a normal healthy cell. In HPV-infected cervical cancers, the oncoproteins E6 and E7 manage to express hTERT (human telomerase reverse transcriptase – the catalytic unit of telomerase enzyme) constitutively to establish replicative immortality. E6 has been reported to activate the hTERT promoter with the help of cellular ubiquitin ligase-E6AP along with c-myc, Sp1, and NFX1 (Liu et al., 2009). NFX1 is a negative repressor of hTERT and thus gets degraded by E6/E6AP to activate hTERT promoter (Gewin et al., 2004; Liu et al., 2009), while the rest two, i.e., c-myc and Sp1 function as a positive regulator and thus get activated by E6 (Liu et al., 2009). E6-mediated constitutive expression of hTERT is also established through epigenetic regulation. Several histone methylases and demethylases are accordingly manipulated to increase the activating H3K4Me3 mark and decrease the repressive H3K9Me2 mark (Zhang et al., 2017). A noteworthy study also showed E6 to increase the serine 2 phosphorylation of RNA polymerase II to promote the transcription of hTERT, which in the absence of E6 is found to remain repressed by USF1/USF2 (McMurray and McCance, 2003).

### Angiogenesis Induction

A major requisite for tumorigenesis is the recruitment of blood circulation to the transforming cells from the existing vasculature, a process termed as “angiogenesis.” This act is maintained by an equilibrium between angiogenesis inducers and angiogenesis inhibitors. E6 and E7 help the HPV infected cells derive nutrition and oxygen from the surrounding tissues through angiogenesis by regulating the expressions and activities of the inducers and inhibitors. The major noteworthy change in the expression of the tumor suppressor p53 and pRb by E6 and E7, respectively, has been found to be linked to the angiogenesis modulators. A microarray analysis by Toussaint-Smith et al. (2004) showed three genes regulated by p53 to change its expression in cells transformed with E6 and E7, including thrombospondin-1, maspin (angiogenesis inhibitors), and VEGF (vascular endothelial growth factor – angiogenesis inducer). Thrombospondin-1 and maspin act as angiogenesis inhibitors and are positively regulated by p53. Thus, since p53 is degraded by E6 successfully, the angiogenesis inhibitors are not functional. VEGF is a well-known angiogenesis inducer, which is negatively regulated by p53 through the HIF-1α, and in the absence of p53 (i.e., mediated by E6), it becomes activated and helps in angiogenesis. IL-8 (Interleukin-8) was also found to increase in the response to E6 and E7 expression in the same report by Toussaint-Smith et al. (2004). IL-8 is known to be a major angiogenesis inducer. VEGF regulation by E6 can occur in a p53-independent mechanism, through the activation of Sp1 transcription factor (Lopez-Ocejo et al., 2000). VEGF promoter contains an AP1 binding site, which can be activated by E7 (Tischer et al., 1991). Recent report by Wang et al. (2014) also showed an upregulation of RRM2 by E7, which functions to induce angiogenesis *via* ROS-ERK1/2-HIF-1α-VEGF.

### Activation of Invasion and Metastasis

E6 and E7 oncogenes have been found to induce the epithelial-to-mesenchymal transition (EMT), a process required for the tumor cells to invade into the bloodstream and metastasize at other place in the body. Using the epithelial MDCK cell line as an *in vitro* model, the study by Kim et al. (2013) showed that ectopic expression of E6/E7 can induce cobblestone-shaped epithelial cell formation from spindle-shaped mesenchymal cells. Moreover, they also showed that E6 and E7 could activate the EMT-inducing transcriptional factors such as Slug, Twist, ZEB1, and ZEB2 followed by an increase in the migratory and invasive potentials of the cells. The EMT markers, like E-cadherin (epithelial cell marker), were found to decrease (D’costa et al., 2012), whereas N-cadherin, fibronectin, and vimentin, the mesenchymal markers, increase in response to E6 and E7 (Hellner et al., 2009). In addition to E6 and E7, E5 has also been reported to upregulate VEGF through EGFR, MEK/ERK1 and 2 along with PI3K/Akt, which induces cell invasion and metastasis (Kim et al., 2006).

## Therapeutics Targeting E6 and E7 Oncoplayers

**Figure 3:**
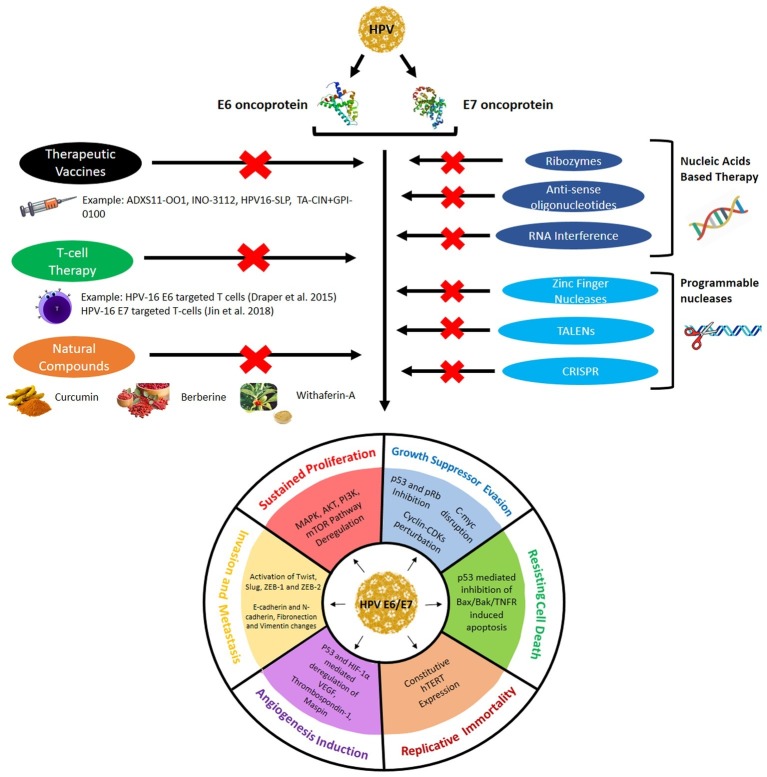
Different therapeutic approaches used to target E6/E7 expression and activity to prevent them from establishing the cervical cancer hallmarks.

As discussed in the earlier segment, E6 and E7 are the major oncoplayers driving the process of HPV-mediated cervical tumorigenesis through the establishment of all the six hallmarks of cancer. Thus, E6 and E7 represent the most effective targets for therapeutics as it can ensure the eradication of all cervical cancer cells by bringing down any or all of the cancer hallmarks. The next section in this review discusses the several approaches used in current clinical research to target E6/E7 for efficacious and safer cervical cancer therapies and has been graphically represented in Figure 3.

### Vaccines Targeting Human Papillomavirus E6/E7

In addition to the prophylactic vaccines, which can activate the body’s immune system to prevent the cervical cancer from occurring, modern clinical research is focusing on the development of therapeutic vaccines, which can be used to treat cancer even at advanced malignant stages. Unlike the preventive vaccines that are developed against the L1 or L2 capsid proteins that get inactivated or deleted during the integration of the HPV genome into the cellular genome, the therapeutic vaccines target oncoproteins E6 and E7, which are expressed for a longer period in the life cycle of the HPV and represent the most significant hallmark of any HPV-infected cell. Hence, the prophylactic vaccines fail to work once the HPV establishes itself, but the therapeutic vaccines can target them even at the most advanced stages. Therapeutic vaccines intend to impart cell-mediated immunity to kill the infected cells instead of introducing neutralizing antibodies into the system like the prophylactic vaccines. Therapeutic vaccines can be categorized based on their source of development, such as live bacterial or viral vector, peptide or protein, and nucleic acid or cell based (reviewed by Chabeda et al., 2018). Several such vaccines are being researched and are currently in clinical trials (phase I/II/III) for cervical cancer. Vaccines such as ADXS11-OO1 (Lm-LLo-E7), INO-3112, HPV16-SLP, and TA-CIN + GPI-0100 have been prepared to target HPV16/18 E6/E7 proteins to treat the advanced stage cervical cancers. They are either undergoing the clinical trials or have completed them. Some of the vaccines have been also prepared specifically for persistent HPV infection and low grade squamous intraepithelial lesion such as PDS0101 and ProCervix, or specifically for cervical intraepithelial lesion (CIN)/high grade squamous intraepithelial lesion like GX-188E, pNGVL4a-CRT/E7 (detox), PepCan + Candin, etc. (reviewed by Yang et al., 2016).

### Genome Editing Technologies to Target E6/E7

With the advent of genome editing tools, the HPV-E6/E7 region of the HPV genome or their respective mRNAs can be specifically targeted to cure cervical cancer. Gene editing techniques used for therapeutic approaches began with the use of antisense oligonucleotides, ribozymes, DNAzymes, siRNA (small interfering RNA), shRNA (short hairpin RNA), and so on. Recent studies have developed stronger techniques to efficiently silent E6/E7 expression, such as zinc finger nucleases (ZFN), transcription activator-like effector nucleases (TALENs), and the clustered regularly interspaced short palindromic repeat-associated nuclease (CRISPR/Cas9) RNA-guided endonuclease. This can help lower the mortality rate of cervical cancer since the advanced cancer stages, which bore the integrated HPV genome and could not be treated, can now be dealt with the advanced gene manipulating technologies.

#### Nucleic Acid-Based Therapy

Nucleic acid-based therapy relies on the use of antisense DNA or RNA molecules to interrupt the expression and/or activity of E6 and E7 genes, which could prevent the progression of such cancer. They include the use of antisense oligonucleotides, catalytic oligonucleotides, and RNA interference. On one hand, the catalytic oligonucleotides (i.e., DNAzymes and Ribozymes) can cleave the target mRNA, while antisense oligonucleotides and small interfering RNA (siRNA) molecules lead to translational inhibition of the target transcripts.

##### Ribozymes

Ribozymes are RNA enzymes, which are capable of hybridizing with the target sequence and cleaving it with the help of its catalytic core. Although they are abundantly present in nature, they can be artificially synthesized to specifically target the disease causing genes, such as E6/E7. Several forms of them are present, but the recombinant ones synthesized are usually hairpin ribozymes or hammerhead ribozymes in nature. For example, studies (Zheng et al., 2002, 2004) showed that hammerhead ribozymes targeting HPV16 E6/E7 transcripts resulted in loss of cell growth *in vitro* and tumor growth in nude mice *in vivo* following apoptosis. Another study showed a ribozyme targeting E6AP in HeLa cells to enhance the apoptotic response and mitomycin-C induced DNA damage (Kim et al., 2003).

##### Antisense Oligonucleotides

Antisense oligonucleotides are around ~20 nucleotides long sequence synthesized complementary to the target mRNA. It results in translational inhibition to prevent the synthesis of disease enhancing protein. These antisense oligonucleotides can either physically disrupt the bonding between the ribosome and the mRNA or activate endogenous Rnase H to cleave the target mRNA. Several attempts have been made to synthesize effective antisense oligonucleotides, such as phosphodiester antisense oligonucleotides, or phosphorothioate analogs followed by methylphosphonate, ethylphosphonate, or peptidic bond modifications in the beginning period, which showed greater susceptibility to nucleases. Hence, modern-day science has come up with highly effective nuclease resistant antisense oligonucleotides, including peptide nucleic acids (PNAs), phosphorodiamidate morpholino oligomers (PMOs), and locked nucleic acids (LNAs), which restore the RNase H recruitment ability. Several studies have reported the use of antisense oligonucleotides targeting different regions of the E6/E7 transcripts with varying delivery methods, which have shown promising results in inhibiting cancer progression both *in vitro* and *in vivo* (reviewed by Bharti et al., 2018). They have been shown to induce apoptosis through upregulation of p53 and pRb, bringing about reduction in the neoplastic appearance. Antisense oligonucleotides targeting regions of E6AP have also shown tumor inhibitory potential (Beer-Romero et al., 1997). A novel study devised an antisense oligonucleotide combined to a photoreactive Ru (ruthenium) complex, which could target E6 in HPV 16 infected SiHa cells, resulting efficient inhibition of cell growth in both monolayer and three-dimensional cultures (Reschner et al., 2013).

##### RNA Interference

RNA interference is a comparatively advanced mode of genome editing, which utilizes double-stranded RNA molecules (termed as small interfering RNA-siRNA or short hairpin RNA-shRNA) to silence the target oncogenes E6 and E7. Several approaches have used this technique to silence E6/E7 either partially or completely, which brought about cell senescence through cellular accumulation of p53, hypophosphorylation of pRb, inhibited cell growth in monolayers, and anchorage-independent growth leading to apoptotic outcomes. Similar results were also observed *in vivo* study wherein the tumor growth reduced upon E6/E7 silencing. HPV16-E7 shRNA when programmed induced degradation of both E6 and E7 transcripts and proteins, resulting in accumulation of cellular p53, p21, and hypophosphorylation of pRb. Ultimately, the loss of E6 and E7 resulted in apoptosis (Sima et al., 2008). siRNA-mediated knockdown of E6/E7 was also found to sensitize the cells to cisplatin-induced cell death (Tan et al., 2012). A review by Togtema et al. (2018) surveyed over 25 experimental data analyzing over 60 synthetically prepared siRNAs targeting HPV16 E6, all of which have been found to be effective in inhibiting the growth of tumor both *in vitro* and *in vivo*. The percentage of E6 knockdown showed variation (~10% to around 80%) depending on the sequence of the oncogenes targeted, the molar concentration of siRNA used, the mode of cellular delivery, the cellular model used for the study, and so on (Togtema et al., 2018).

Thus, all the forms of nucleic acid-based therapies when target the oncogenes E6 and E7 bring about efficient reduction in cervical cancer progression through similar pathways of cell growth inhibition and apoptosis.

#### Genome Editing With Programmable Nucleases

Programmable nucleases including Zinc-finger nucleases (ZFNs), Transcription activator-like effector nucleases (TALENs), and CRISPR-Cas9 systems can be used to target the disease causing oncogenes like E6 and E7 to completely abrogate their activity in order to restrict and recede the cervical tumor growth. Genome editing using any of the above procedures involves the use of sequence-specific DNA binding domains merged to non-specific DNA cleavage units, which lead to precise changes in the gene of interest. Efficiency of the technology used depends on the sequence specificity and affinity of the nucleases, which can be programmed accordingly.

##### Zinc-Finger Nucleases

Zinc-finger nucleases (ZFNs) are artificial endonucleases programmed by fusion of DNA-binding zinc-finger proteins with FokI DNA-cleavage domain (Kim et al., 1996). When two artificially programmed ZFNs dimerize upon binding to the DNA, they form an active FokI nuclease, which can cleave the target sequence through double-stranded break induction. The double-stranded breaks introduced can either lead to cell death through programmed pathway of apoptosis or lead to activation of DNA repair mechanism. Usually non-homologous end joining is recruited, which introduces several mutations in the genes targeted. These result in regression of the disease caused by the target genes. ZFNs were first used against HPV E2 gene, which prevented the virus from replicating within the host cell (Mino et al., 2009, 2013). Later, ZFNs were customized to target the HPV-E7 gene, which successfully disrupted the HPV DNA, inhibited the growth of the HPV 16/18 positive cervical cancer cells *in vitro*, and were found to undergo apoptosis. They were further proved to be clinically more efficient as they could also establish their therapeutic effect in xenograft mouse model (Ding et al., 2014). Shankar et al. (2018) reported that they could not successfully inhibit E6 expression and activity using ZFNs as no matching target sequence could be obtained using publicly available computer software. Thus, they used TALENs instead of the same purpose.

##### Transcription Activator-Like Effector Nuclease

Transcription activator-like effector nucleases (TALENs) are programmable fusion proteins made up of a N-terminal translocation region, central repeat segments of 33–35 residues that can bind to the DNA in a sequence-specific manner and a C-terminal unit that bears the nuclear localization signal (NLS) along with the FokI endonuclease 8 (Wyman and Kanaar, 2006; Gaj et al., 2013; Qu et al., 2013). In 2015, a team led by Hu et al. (2015) reported for the first time TALEN-mediated genome editing of the HPV oncogenes E6/E7 can successfully inhibit tumor growth, induce apoptosis, and reduce tumorigenic capability of HPV-infected cells both *in vitro* and *in vivo*. They found similar outcomes in both HPV16 (SiHa) and HPV18 (HeLa) infected cells *in vitro*. Next Shankar et al. (2017) screened for TALEN pairs to target E7 exon of the HPV genome using SAPTA software. The TALEN pair that was sequenced and synthesized targeted 44th position to 103rd nucleotide encompassing the start region of exon I of E7, and it had 19 binding sites on either side of a spacer region. The selected TALEN pairs effectively edited the HPV E7 genome of HPV16-infected SiHa cells as found in the transcript and protein level. It resulted in a corresponding increase in the amount of pRb and a decrease in p14ARF, the downstream targets following a necrotic pathway of cell death as shown through the upregulation of RIP-1, Cyclophilin A, and LDH-A. In the due course, another study by Shankar et al. (2018) reported that TALEN-mediated editing of HPV16 E6 did not yield any editing activity, while E7 could be successfully knocked down in HPV16 infected CasKi cells. E6-targeted TALEN was composed of 18 modules on both the arms spaced by a 19 nucleotide region, while the E7 targeting TALEN contained 18 modules on both the ends separated by a 21 nucleotide gap. Hence, programmable nucleases should be designed to have efficient knockout capability depending on the sequence specificity and so that the off-target effects are minimized in order to establish their immense therapeutic potential.

##### Clustered Regularly Interspaced Short Palindromic Repeats-Cas9

Clustered regularly interspaced short palindromic repeats (CRISPR)-Cas9 is a novel genome editing tool originally observed as a part of adaptive immune system in bacterial systems to fight the foreign nucleic acids (Hsu et al., 2014; Makarova et al., 2015; Wright et al., 2016). Unlike ZFN and TALEN systems wherein protein domains need to be manipulated, CRISPR-Cas systems are comparatively simpler as it involves the use of a single-guide RNA, which can guide the endonuclease Cas9 to introduce double-stranded breaks in the specific target gene, leading to the knockdown of the gene through the use of cellular host repair machinery. Thus, therapeutically it has evolved to gain the scientific attention in the treatment of HPV-infected cancer progression. Several studies have reported the use of CRISPR-Cas9 mediated E6/E7 silencing to regress the cervical cancer progression. The guide RNAs can be programmed accordingly to target specific exon segments of the oncogenes E6/E7. Thus, CRISPR-Cas9 mediated silencing of both E6 and E7 oncogenes in HPV 16 infected SiHa/CasKi cells and HPV 18 infected HeLa cells led to growth inhibition, cell cycle arrest, and cell death through p53 and pRb restoration (Hsu et al., 2014; Kennedy et al., 2014). Later, Yoshiba et al. (2018) tried targeting E6 using CRISPR-Cas9 system delivered through AAV type 2 vector and were successful in introducing several mutations in the oncogene E6. E6 when silenced led to reduced tumor growth both *in vitro* and *in vivo* along with an increase in the p53 status finally leading to apoptotic mode of cell death.

Although these high-end genome editing technologies come with immense therapeutic potential but are not easily affordable. Moreover, the delivery systems also remain a major obstacle due to the negative charge, large size, and low membrane penetration (Bharti et al., 2018). Since cervical cancer mostly affects the women from underdeveloped population sectors, the applicability of these genome editing techniques in clinical therapies remains a major question. Search for efficient low-cost therapeutics is still a major research focus to help the people with poor financial strata, i.e., the ones usually targeted with cervical cancer.

## Immunotherapeutic Targeting of E6/E7

An ideal tumor progressive environment requires inactivation of the body’s natural immune system, which includes lower count of T cells and their suppressed activity and changes in cytokine expression and faulty antigen presentation (Varilla et al., 2013; Sun et al., 2014; Mandal et al., 2016). E6- and E7-specific cellular and humoral immune responses are clinically successful owing to the continuous activity of these oncogenes throughout the disease progression. Synthetic HPV 16/18 E6 and E7 DNA sequences were introduced to activate the immune system using a novel plasmid VGX-3100 (Inovio Pharmaceuticals; Bagarazzi et al., 2012; Trimble et al., 2015). Following this, a pilot study introduced CD8+ T cells in patients, which resulted in histopathological regression and HPV 16/18 clearance too (Bagarazzi et al., 2012; Trimble et al., 2015). A combination of synthetic plasmids termed as MEDI0457 (earlier called as INO-3112) was created to target HPV16 and HPV18 E6/E7 antigens along with a recombinant IL-12 (interleukin-12) as a molecular adjuvant (INO-9012). IL-12 is known to promote the maturation and activity of T cells, which enhances the immune activity. MEDI0457 was found to induce long-lived antibody responses, robust HPV-specific interferon-gamma production from T cells, and antigen-specific cytotoxic T cell production. Thus, such improved cellular and humoral immune response when combined with other treatment methods reduced local recurrence and rate of metastasis. Jin et al. (2018) also reported that T cells can be genetically engineered to target HPV16 E7, which can lead to regression of HPV16-infected cervical cancers in xenografted mouse model. HPV16 E6 can also be targeted through genetically engineered T cells having similar outcome (Draper et al., 2015). Complete regression of metastatic cervical cancer has also been reported in response to a single infiltration of T cells targeted for HPV16 E6/E7 (Stevanovic et al., 2015). Adoptive T-cell therapy is a promising avenue; the use of genetic programming to target the HPV oncoplayers using the T cells can widen the scope of immunotherapy by helping in targeting the oncogenes specifically with no side effects.

## Phytotherapy Targeting E6 and E7

Phytotherapeutic approach relies on the use of natural products for the treatment of several deadly cancer forms, one of them being cervical cancer. The many different forms of anticancer drugs used in modern-day chemotherapies are either directly obtained from natural sources or modified versions of them, including the well-known vincristine, podophyllotoxin, camptothecin, vinblastine, paclitaxel, docetaxel, homoharringtonine, and so on. In addition to this, several plant compounds or plant extracts have shown promising anticancer effects in cervical cancer cell lines *in vitro* and *in vivo*. Among them, many different natural compounds were found to directly abrogate HPV-E6/E7 activity, which have been listed in Table 1. Such compounds are important drug candidates for research since they have the potential to directly knockdown the oncoplayers and help in cervical cancer regression. Moreover, a series of flavonoids have been identified that can bind to E6 and inhibit the p53 degradation resulting in decreased viability of HPV infected cervical cancer cells (Cherry et al., 2013). In addition to luteolin, several novel flavonoids were also identified in the process with a promising therapeutic role in cervical cancer. Several plant extracts and products like latex from *Ficus carica*, seed oil from flax, or the stem extracts of *Cudrania tricuspidata* also showed anti-oncogenic potential as they could bring down the expression of E6 and E7 transcripts and proteins, with a concomitant apoptotic conclusion (Kwon et al., 2016; Deshpande et al., 2019; Ghanbari et al., 2019). The use of natural products promises a comparatively safer alternative therapeutic approach to cervical cancer. Natural product-based remedy offers easier, abundantly available, inexpensive method of treatment in comparison to the genome editing technologies or the immunotherapeutic method. The prophylactic and the therapeutic vaccines, or the T-cell-based therapies, or the several modes of genome editing techniques are too expensive to be afforded by the financially poor section of population, and the most noteworthy fact points to these economically backward people as the most commonly targeted group of people with cervical cancer, as discussed previously.

**Table 1:** Phytocompounds with anti-oncogenic activity for E6 and E7.

S. no.	Phytocompound	Natural source	Cell type/model	Study observations	Ref.
1	Curcumin	*Curcuma longa* (rhizome)	HeLa, SiHa, and C33A	Inhibition of E6 and E7 transcripts and protein, prevention of NF-κβ and AP1 translocation, and apoptosis induction	Divya and Pillai (2006)
2	Berberine	*Berberis* sp. (rhizome/bark)	HeLa and SiHa	Repression of E6 and E7 with a concomitant increase in p53 and pRb activates AP1 and induces apoptosis. Suppresses hTERT expression.	Mahata et al. (2011)
3	Jaceosidin	*Artemisia argyi* (leaves)	SiHa and CasKi	Inhibited binding of E6 and p53 and E7 and pRb	Lee et al. (2005)
4	Wogonin	*Scutellaria baicalensis*	SiHa and CasKi	Promotes intrinsic apoptosis through suppression of E6 and E7 and increase in p53 and pRb	Kim et al. (2013)
5	Nordihydroguaiaretic acid	*Scutellaria baicalensis*	SiHa	Inhibition of E6 expression and upregulation of p53 and p21	Gao et al. (2010)
6	Tanshinone IIA	*Salvia* sp.	HeLa, SiHa, CasKi, and C33A	Downregulates E6 and E7, induces apoptosis and cell cycle arrest, inhibits tumor growth *in vivo*	Munagala et al. (2014)
7	Withaferin A	*Withania somnifera*	CasKi	Apoptosis through E6 and E7 repression; inhibition of tumor growth *in vivo*	Munagala et al. (2011)

## Conclusion

E6 and E7 as correctly coined “HPV oncoplayers” are the major driving force for cervical carcinogenesis. They are responsible from the initiation point of tumor development including the maintenance of continuous proliferative signaling, the escape of tumor suppressors, and activation of telomerase to the induction of angiogenesis and invasion to metastatic stages. These are the six primary hallmarks of any form of cancer established by Hanahan and Weinberg (2000). E6 and E7 singly take in charge of all the six hallmarks to establish and help in the successful progression of cervical cancer. Thus, this review tried to incorporate the significance of these HPV oncogenes in all the spheres of cancer cell activity and also included their therapeutic role. Since, these oncogenes are the major carcinogenic factors, targeting cervical cancer cells specifically could be achieved with the help of E6 and E7 targeting. This makes them the most effective drug candidates for HPV-infected cancers, including cervical cancer. Several forms of therapies have been studied and tried using E6 and E7 targeting, all of which have their own merits and demerits. Combinatorial approach is the newest form of clinical practice, which could result in better ways to combat the high-mortality index of cervical cancer worldwide.

## Author Contributions

AP and RK designed the structure of the article, researched through the experimental publications, and discussed the conclusive idea of the review article. AP wrote and edited the manuscript. RK reviewed the manuscript before submission.

### Conflict of Interest

The authors declare that the research was conducted in the absence of any commercial or financial relationships that could be construed as a potential conflict of interest.

## References

[ref1] Aguilar-Lemarroy A. Gariglio P. Whitaker N. J. Eichhorst S. T. Zur Hausen H. Krammer P. H.. (2002). Restoration of p53 expression sensitizes human papillomavirus type 16 immortalized human keratinocytes to CD95-mediated apoptosis. Oncogene 21, 165–175. 10.1038/sj.onc.1204979, PMID: 11803460

[ref2] American Cancer Society (2018). Cancer facts and figures 2018. Atlanta, GA: American Cancer Society Available at: https://www.cancer.org/cancer/cervical-cancer/causes-risks-prevention/risk-factors.html (Accessed September 15, 2019).

[ref3] Bagarazzi M. L. Yan J. Morrow M. P. Shen X. Parker R. L. Lee J. C.. (2012). Immunotherapy against HPV16/18 generates potent TH1 and cytotoxic cellular immune responses. Sci. Transl. Med. 4:155ra138. 10.1126/scitranslmed.3004414, PMID: 23052295 PMC4317299

[ref4] Barbosa M. S. Edmonds C. Fisher C. Schiller J. T. Lowy D. R. Vousden K. H. (1990). The region of the HPV E7 oncoprotein homologous to adenovirus E1a and SV40 large T antigen contains separate domains for Rb binding and casein kinase II phosphorylation. EMBO J. 9, 153–160. 10.1002/j.1460-2075.1990.tb08091.x, PMID: 2153075 PMC551641

[ref5] Beer-Romero P. Glass S. Rolfe M. (1997). Antisense targeting of E6AP elevates p53 in HPV-infected cells but not in normal cells. Oncogene 14, 595–602. 10.1038/sj.onc.1200872, PMID: 9053858

[ref420] Bernard H. U. Burk R. D. Chen Z. van Doorslaer K. zur Hausen H. de Villiers E. M. (2010). Classification of Papillomaviruses (PVs) based on 189 PV types and proposal of taxonomic amendments. Virology 401, 70–79. 10.1016/j.virol.2010.02.002, PMID: 20206957 PMC3400342

[ref6] Bharti A. C. Singh T. Bhat A. Pande D. Jadli M. (2018). Therapeutic strategies for human papillomavirus infection and associated cancers. Front. Biosci. 10, 15–73. 10.2741/e808, PMID: 28930604

[ref7] Borbély A. A. Murvai M. Kónya J. Beck Z. Gergely L. Li F. (2006). Effects of human papillomavirus type 16 oncoproteins on survivin gene expression. J. Gen. Virol. 87, 287–294. 10.1099/vir.0.81067-0 16432013

[ref8] Bouvard V. Baan R. Straif K. Grosse Y. Secretan B. Ghissasi F. E.. (2009). A review of human carcinogens-part B: biological agents. Lancet Oncol. 10, 321–322. 10.1016/S1470-2045(09)70096-8, PMID: 19350698

[ref9] Boyer S. N. Wazer D. E. Band V. (1996). E7 protein of human Papillomavirus16 induces degradation of retinoblastoma protein through the ubiquitin-proteasome pathway. Cancer Res. 56, 4620–4624. PMID: 8840974

[ref10] Bray F. Ferlay J. Soerjomataram I. Siegel L. Torre L. A. Jemal A. (2018). Global cancer statistics 2018: GLOBOCAN estimates of incidence and mortality worldwide for 36 cancers in 185 countries. CA Cancer J. Clin. 68, 394–424. 10.3322/caac.21492, PMID: 30207593

[ref11] Bretones G. Delgado M. D. Leon J. (2015). Myc and cell cycle control. Biochim. Biophys. Acta 1849, 506–516. 10.1016/j.bbagrm.2014.03.013, PMID: 24704206

[ref58] Burk R. D. Chen Z. Saller C. Tarvin K. Carvalho A. L. Scapulatempo-Neto C.. (2017). Integrated genomic and molecular characterization of cervical cancer. Nature 543, 378–384. 10.1038/nature21386, PMID: 28112728 PMC5354998

[ref12] Chabeda A. Yanez R. J. R. Lamprecht R. Meyers A. E. Rybicki E. P. Hitzeroth I. I. (2018). Therapeutic vaccines for high risk HPV-associated diseases. Papillomavirus Res. 5, 46–58. 10.1016/j.pvr.2017.12.006, PMID: 29277575 PMC5887015

[ref13] Chakrabarti O. Veeraraghavalu K. Tergaonkar V. Liu Y. Androphy E. J. Stanley M. A.. (2004). Human papillomavirus type 16 E6 amino acid 83 variants enhance E6-mediated MAPK signalling and differentially regulate tumorigenesis by notch signalling and oncogenic Ras. J. Virol. 78, 5934–5945. 10.1128/JVI.78.11.5934-5945.2004, PMID: 15140991 PMC415794

[ref14] Cherry J. J. Rietz A. Malinkevich A. Liu Y. Xie M. Bartolowits M.. (2013). Structure based identification and characterisation of flavonoids that disrupt human papillomavirus-16 E6 function. PLoS One 8:e84506. 10.1371/journal.pone.0084506, PMID: 24376816 PMC3871595

[ref15] D’costa Z. J. Jolly C. Androphy E. J. Mercer A. Matthews C. M. Hibma M. H. (2012). Transcriptional repression of E-cadherin by human papillomavirus type 16 E6. PLoS One 7:e48954. 10.1371/journal.pone.0048954, PMID: 23189137 PMC3506579

[ref16] de Villiers E. M. Fauquet C. Broker T. R. Bernard H. U. Hausen H. Z. (2004). Classification of papillomaviruses. Virology 324, 17–27. 10.1016/j.virol.2004.03.033, PMID: 15183049

[ref17] Deshpande R. Raina P. Shinde K. Mansara P. Karandikar M. Kaul-Ghanekar R. (2019). Flax seed oil reduced tumour growth, modulated immune responses and decreased HPV E6 and E7 oncoprotein expression in a murine model of ectopic cervical cancer. Prostaglandins Other Lipid Mediat. 143:106332. 10.1016/j.prostaglandins.2019.04.002, PMID: 30959179

[ref18] Ding W. Hu Z. Zhu D. Jiang X. Yu L. Wang X.. (2014). Zinc finger nucleases targeting the human papillomavirus E7 oncogene induce E7 disruption and a transformed phenotype in HPV16/18-positive cervical cancer cells. Clin. Cancer Res. 20, 6495–6503. 10.1158/1078-0432.CCR-14-0250, PMID: 25336692

[ref19] Divya C. S. Pillai M. R. (2006). Antitumor action of curcumin in human papillomavirus associated cells involves downregulation of viral oncogenes, prevention of NFkB and AP-1 translocation, and modulation of apoptosis. Mol. Carcinog. 45, 320–332. 10.1002/mc.20170, PMID: 16526022

[ref20] Doorbar J. Egawa N. Griffin H. Kranjec C. Murakami I. (2016). Human papillomavirus molecular biology and disease association. Rev. Med. Virol. 25, 2–23. 10.1002/rmv.1822 PMC5024016 25752814

[ref21] Draper L. M. Kwong M. L. Gros A. Stevanovic S. Tran E. Kerkar S.. (2015). Targeting of HPV16+ epithelial cancer cells by TCR gene engineered T cells directed against E6. Clin. Cancer Res. 21, 4431–4439. 10.1158/1078-0432.CCR-14-3341, PMID: 26429982 PMC4603283

[ref22] Egawa N. Doorbar J. (2017). The low risk papillomaviruses. Virus Res. 231, 119–127. 10.1016/j.virusres.2016.12.017, PMID: 28040475

[ref23] Eichten A. Rud D. S. Grace M. Piboonniyom S. O. Zacny V. Munger K. (2004). Molecular pathways executing the “trophic sentinel” response in HPV16 E7- expressing normal human diploid fibroblasts upon growth factor deprivation. Virology 319, 81–93. 10.1016/j.virol.2003.11.008, PMID: 14967490

[ref24] Filippova M. Song H. Connolly J. L. Dermody T. S. Duerksen-Hughes P. J. (2002). The human papillomavirus16 E6 protein binds to tumor necrosis factor (TNF) R1 and protects cells from TNF-induced apoptosis. J. Biol. Chem. 277, 21730–21739. 10.1074/jbc.M200113200, PMID: 11934887

[ref25] Gaj T. Gersbach C. A. Barbas C. F. III (2013). ZFN, TALEN, and CRISPR/ Cas-based methods for genome engineering. Trends Biotechnol. 31, 397–405. 10.1016/j.tibtech.2013.04.004, PMID: 23664777 PMC3694601

[ref26] Gao P. Zhai F. Guan L. Zheng J. (2010). Nordihydroguaiaretic acid inhibits growth of cervical cancer SiHa cells by up-regulating p21. Oncol. Lett. 2, 123–128. 10.3892/ol.2010.205 22870140 PMC3412500

[ref27] Garnett T. O. Duerksen-Hughes P. J. (2006). Modulation of apoptosis by human papillomavirus (HPV) oncoproteins. Arch. Virol. 151, 2321–2335. 10.1007/s00705-006-0821-0, PMID: 16862386 PMC1751433

[ref28] Gewin L. Myers H. Kiyono T. Galloway D. A. (2004). Identification of a novel telomerase repressor that interacts with the human papillomavirus type-16 E6/E6-AP complex. Genes Dev. 18, 2269–2282. 10.1101/gad.1214704, PMID: 15371341 PMC517520

[ref29] Ghanbari A. Gresley A. L. Naughton D. Kuhnert N. Sirbu D. Ashrafi G. H. (2019). Biological activities of *Ficus carica* latex for potential therapeutics in human papillomavirus (HPV) related cervical cancers. Sci. Rep. 9:1013. 10.1038/s41598-018-37665-6, PMID: 30705373 PMC6355798

[ref30] Hanahan D. Wienberg R. A. (2000). Hallmarks of cancer. Cell 100, 57–70. 10.1016/S0092-8674(00)81683-9, PMID: 10647931

[ref32] Hellner K. Mar J. Fang F. Quackenbush J. Munger K. (2009). HPV 16 E7 oncogene expression in normal human epithelial cells causes molecular changes indicative of an epithelial to mesenchymal transition. Virology 391, 57–63. 10.1016/j.virol.2009.05.036, PMID: 19552933 PMC2728214

[ref33] Hsu P. D. Lander E. S. Zhang F. (2014). Development and applications of CRISPR-Cas9 for genome engineering. Cell 157, 1262–1278. 10.1016/j.cell.2014.05.010, PMID: 24906146 PMC4343198

[ref34] Hu Z. Ding W. Zhu D. Yu L. Jiang X. Wang X.. (2015). TALEN-mediated targeting of HPV oncogenes ameliorates HPV-related cervical malignancy. J. Clin. Invest. 125, 425–436. 10.1172/JCI78206, PMID: 25500889 PMC4382249

[ref35] Jabbar S. F. Abrams L. Glick A. Lambert P. F. (2009). Persistence of high-grade cervical dysplasia and cervical cancer requires the continuous expression of the human papillomavirus type 16 E7 oncogene. Cancer Res. 69, 4407–4414. 10.1158/0008-5472.CAN-09-0023, PMID: 19435895 PMC3006677

[ref423] Jin B. Y. Campbell T. E. Draper L. M. Stevanovic S. Weissbrich B. Yu Z.. (2018). Engineered T cells targeting E7 mediate regression of human papillomavirus cancers in a murine model. J. Clin. Investig. Insight 3, pii: 99488. 10.1172/jci.insight.99488, PMID: 29669936 PMC5931134

[ref36] Kelley M. L. Keiger K. E. Lee C. J. Huibregtse J. M. (2005). The global transcriptional effects of the human papillomavirus E6 protein in cervical carcinoma cell lines are mediated by the E6AP ubiquitin ligase. J. Virol. 79, 3737–3747. 10.1128/JVI.79.6.3737-3747.2005, PMID: 15731267 PMC1075713

[ref37] Kennedy E. M. Kornepati A. V. R. Goldstein M. Bogerd H. P. Poling B. C. Whisnant A. W. (2014). Inactivation of human papillomavirus E6 or E7 gene in cervical carcinoma cells by using a bacterial CRISPR/Cas RNA-guided endonuclease. J. Virol. 88, 11965–11972. 10.1128/JVI.01879-14, PMID: 25100830 PMC4178730

[ref38] Khan S. H. Wahl G. M. (1998). P53 and pRb prevent rereplication in response to microtubule inhibitors by mediating a reversible G1 arrest. Cancer Res. 58, 396–401. PMID: 9458079

[ref39] Kim M. S. Bak Y. Park Y. S. Lee D. H. Kim J. H. Kang J. W.. (2013). Wogonin induces apoptosis by suppressing E6 and E7 expressions and activating intrinsic signalling pathways in HPV16 cervical cancer cells. Cell Biol. Toxicol. 29, 259–272. 10.1007/s10565-013-9251-4, PMID: 23955116

[ref40] Kim Y. Cairns M. J. Marouga R. Sun L. Q. (2003). E6AP gene suppression and characterization with in vitro selected hammerhead ribozymes. Cancer Gene Ther. 10, 707–716. 10.1038/sj.cgt.7700623, PMID: 12944990

[ref41] Kim Y. G. Cha J. Chandrasegaran S. (1996). Hybrid restriction enzymes: zinc finger fusions to Fok I cleavage domain. Proc. Natl. Acad. Sci. USA 93, 1156–1160. 10.1073/pnas.93.3.1156 8577732 PMC40048

[ref42] Kim S. H. Juhnn Y. S. Kang S. Park S. W. Sung M. W. Bang Y. J.. (2006). Human papillomavirus 16 E5 up-regulates the expression of vascular endothelial growth factor through the activation of epidermal growth factor receptor, MEK/ERK1, 2 and PI3K/Akt. Cell. Mol. Life Sci. 63, 930–938. 10.1007/s00018-005-5561-x, PMID: 16596339 PMC11136283

[ref43] Kwon S. B. Kim M. J. Yang J. M. Lee H. P. Hong J. T. Jeong H. S.. (2016). *Cudrania tricuspidata* stem extract induces apoptosis via the extrinsic pathway in SiHa cervical cancer cells. PLoS One 11:e0150235. 10.1371/journal.pone.0150235, PMID: 26960190 PMC4784787

[ref44] LaVigne A. W. Triedman S. A. Randall T. C. Trimble E. L. Viswanathan A. N. (2017). Cervical cancer in low and middle income countries: addressing barriers to radiotherapy delivery. Gynecol. Oncol. Rep. 22, 16–20. 10.1016/j.gore.2017.08.004 28948205 PMC5602511

[ref45] Lee H. G. Yu K. A. Oh W. K. Baeg T. W. Oh H. C. Ahn J. S.. (2005). Inhibitory effect of jaceosidin isolated from *Artemisia argyi* on the function of E6 and E7 oncoproteins of HPV16. J. Ethnopharmacol. 98, 339–343. 10.1016/j.jep.2005.01.054, PMID: 15814270

[ref46] Liu X. Dakic A. Zhang Y. Dai Y. Chen R. Schlegel R. (2009). HPV E6 protein interacts physically and functionally with the cellular telomerase complex. Proc. Natl. Acad. Sci. USA 106, 18780–18785. 10.1073/pnas.0906357106 19843693 PMC2773972

[ref422] Liu X. Disbrow G. L. Yuan H. Tomaic V. Schlegel R. (2007). Myc and Human Papillomavirus Type 16 E7 Genes Cooperate to Immortalize Human Keratinocytes. J. Virol. 81, 12689–12695. 10.1128/JVI.00669-07, PMID: 17804506 PMC2168992

[ref47] Lopez-Ocejo O. Viloria-Petit A. Bequet-Romero M. Mukhopadhyay D. Rak J. Kerbel R. S. (2000). Oncogenes and tumor angiogenesis: the HPV16 E6 oncoprotein activates the vascular endothelial growth factor (VEGF) gene promoter in a p53 independent manner. Oncogene 19, 4611–4620. 10.1038/sj.onc.1203817, PMID: 11030150

[ref48] Mahata S. Bharti A. C. Shukla S. Tyagi A. Husain S. A. Das B. C. (2011). Berberine modulates AP-1 activity to suppress HPV transcription and downstream signalling to induce growth arrest and apoptosis in cervical cancer cells. Mol. Cancer 10:39. 10.1186/1476-4598-10-39, PMID: 21496227 PMC3098825

[ref49] Makarova K. S. Wolf Y. I. Alkhnbasi O. S. Costa F. Shah S. A. Saunders S. J.. (2015). An updated evolutionary classification of CRISPR-Cas systems. Nat. Rev. Microbiol. 13, 722–736. 10.1038/nrmicro3569, PMID: 26411297 PMC5426118

[ref50] Mandal R. Şenbabaoğlu Y. Desrichard A. Havel J. J. Dalin M. G. Riaz N. (2016). The head and neck cancer immune landscape and its immunotherapeutic applications. JCI Insight 1:e89829. 10.1172/jci.insight.89829 27777979 PMC5070962

[ref51] McIntyre M. C. Frattini M. G. Grossman S. R. Laimins L. A. (1993). Human papillomavirus type 18 E7 protein requires intact Cys-X-X-Cys motifs for zinc binding, dimerization, and transformation but not for Rb binding. J. Virol. 67, 3142–3150. PMID: 8497045 10.1128/jvi.67.6.3142-3150.1993 PMC237652

[ref52] McLaughlin-Drubin M. E. Park D. Munger K. (2013). Tumour suppressor p16^INK4A^ is necessary for survival of cervical carcinoma cell lines. Proc. Natl. Acad. Sci. USA 110, 16175–16180. 10.1073/pnas.1310432110 24046371 PMC3791710

[ref53] McMurray H. R. McCance D. J. (2003). Human papillomavirus type 16 E6 activates TERT gene transcription through induction of c-Myc and release of USF-mediated repression. J. Virol. 77, 9852–9861. 10.1128/JVI.77.18.9852-9861.2003, PMID: 12941894 PMC224601

[ref54] Mino T. Aoyama Y. Sera T. (2009). Efficient double stranded DNA cleavage by artificial zinc-finger nucleases composed of one zinc-finger protein and a single chain FokI dimer. J. Biotechnol. 140, 156–161. 10.1016/j.jbiotec.2009.02.004, PMID: 19428709

[ref55] Mino T. Mori T. Aoyama Y. Sera T. (2013). Gene and protein delivered zinc finger staphylococcal nuclease hybrid for inhibition of DNA replication of human papillomavirus. PLoS One 8:e56633. 10.1371/journal.pone.0056633, PMID: 23437192 PMC3577882

[ref56] Munagala R. Aqil F. Jeyabalan J. Gupta R. C. (2014). Tanshinone IIA inhibits viral oncogene expression leading to apoptosis and inhibition of cervical cancer. Cancer Lett. 356, 536–546. 10.1016/j.canlet.2014.09.037 25304375

[ref57] Munagala R. Kauser H. Munjal C. Gupta R. C. (2011). Withaferin A induces p53-dependent apoptosis by repression of HPV oncogenes and upregulation of tumor suppressor proteins in human cervical cancer cells. Carcinogenesis 32, 1697–1705. 10.1093/carcin/bgr192 21859835

[ref59] Neveu G. Cassonnet P. Vidalain P. O. Rolloy C. Mendoza J. Jones L.. (2012). Comparative analysis of virus–host interactomes with a mammalian high-throughput protein complementation assay based on *Gaussia princeps* luciferase. Methods 58, 349–359. 10.1016/j.ymeth.2012.07.029, PMID: 22898364 PMC3546263

[ref60] Nguyen M. Song S. Liem A. Androphy E. Liu Y. Lambert P. F. (2002). A mutant of human papillomavirus type 16 E6 deficient in binding alpha-helix partners displays reduced oncogenic potential in vivo. J. Virol. 76, 13039–13048. 10.1128/JVI.76.24.13039-13048.2002, PMID: 12438630 PMC136691

[ref61] Nomine Y. Masson M. Charbonnier S. Zanier K. Ristriani T. Deryckere F.. (2006). Structural and functional analysis of E6 oncoprotein: insights in the molecular pathways of human papillomavirus-mediated pathogenesis. Mol. Cell 21, 665–678. 10.1016/j.molcel.2006.01.024, PMID: 16507364

[ref64] Ohlenschlager O. Seiboth T. Zengerling H. Briese L. Marchanka A. Ramachandran R.. (2006). Solution structure of the partially folded high-risk human papilloma virus 45 oncoprotein E7. Oncogene 25, 5953–5959. 10.1038/sj.onc.1209584, PMID: 16636661

[ref65] Peter M. Rosty C. Couturier J. Radvanyi F. Teshima H. Sastre-Garau X. (2006). MYC activation associated with the integration of HPV DNA at the MYC locus in genital tumors. Oncogene 25, 5985–5993. 10.1038/sj.onc.1209625, PMID: 16682952

[ref66] Pflaum J. Schlosser S. Muller M. (2014). P53 family and cellular stress responses in cancer. Front. Oncol. 4:285. 10.3389/fonc.2014.00285, PMID: 25374842 PMC4204435

[ref67] Phelps W. C. Yee C. L. Münger K. Howley P. M. (1988). The human papillomavirus type 16 E7 gene encodes transactivation and transformation functions similar to those of adenovirus E1A. Cell 53, 539–547. 10.1016/0092-8674(88)90570-3, PMID: 2836062

[ref68] Qu X. Wang P. Ding D. Li L. Wang H. Ma L.. (2013). Zinc-finger-nucleases mediate specific and efficient excision of HIV-1 proviral DNA from infected and latently infected human T cells. Nucleic Acids Res. 41, 7771–7782. 10.1093/nar/gkt571, PMID: 23804764 PMC3763554

[ref62] Rashid N. N. Rothan H. A. Yusoff M. S. M. (2015). The association of mammalian DREAM complex and HPV 16 E7 proteins. Am. J. Cancer Res. 5, 3525–3533. PMID: 26885443 PMC4731628

[ref63] Rashid N. N. Yusof R. Watson R. J. (2011). Disruption of repressive p130-DREAM complexes by human papillomavirus 16 E6/E7 oncoproteins is required for cell-cycle progression in cervical cancer cells. J. Gen. Virol. 92, 2620–2627. 10.1099/vir.0.035352-0, PMID: 21813705

[ref69] Reschner A. Bontems S. Le Gac S. Lambermont J. Marcelis L. Defrancq E.. (2013). Ruthenium oligonucleotides, targeting HPV 16 E6 oncogene, inhibit the growth of cervical cancer cells under illumination by a mechanism involving p53. Gene Ther. 20, 435–443. 10.1038/gt.2012.54, PMID: 22809997

[ref70] Sadasivam S. DeCaprio J. A. (2013). The DREAM complex: master coordinator of cell cycle dependent gene expression. Nat. Rev. Cancer 13, 585–595. 10.1038/nrc3556, PMID: 23842645 PMC3986830

[ref71] Scheffner M. Huibregtse J. M. Vierstra R. D. Howley P. M. (1993). The HPV16 E6 and E6-AP complex functions as a ubiquitin-protein ligase in the ubiquitination of p53. Cell 75, 495–505. 10.1016/0092-8674(93)90384-3, PMID: 8221889

[ref72] Scheffner M. Werness B. A. Huibregtse J. M. Levine A. J. Howley P. M. (1990). The E6 oncoprotein encoded by human papillomavirus types 16 and 18 promotes the degradation of p53. Cell 63, 1129–1136. 10.1016/0092-8674(90)90409-8, PMID: 2175676

[ref73] Serrano B. Brotons M. Bosch F. X. Bruni L. (2017). Epidemiology and burden of HPV related disease. Best Pract. Res. Clin. Obstet. Gynaecol. 47, 14–26. 10.1016/j.bpobgyn.2017.08.006 29037457

[ref74] Shankar S. Prasad D. Sanawar R. Das A. V. Pillai M. R. (2017). TALEN based HPV-E7 editing triggers necrotic cell death in cervical cancer cells. Sci. Rep. 7:5500. 10.1038/s41598-017-05696-0, PMID: 28710417 PMC5511212

[ref75] Shankar S. Sreekumar A. Prasad D. Das A. V. Pillai M. R. (2018). Genome editing of oncogenes with ZFNs and TALENs: caveats in nuclease design. Cancer Cell Int. 18:169. 10.1186/s12935-018-0666-0, PMID: 30386178 PMC6198504

[ref76] Sima N. Wang W. Kong D. Deng D. Xu Q. Zhou J.. (2008). RNA interference against HPV16 E7 oncogene leads to viral E6 and E7 expression in cervical cancer cells and apoptosis via upregulation of Rb and p53. Apoptosis 13, 273–281. 10.1007/s10495-007-0163-8, PMID: 18060502

[ref77] Spangle J. M. Munger K. (2010). The human papillomavirus type 16 E6 oncoprotein activates mTORC1 signaling and increases protein synthesis. J. Virol. 84, 9398–9407. 10.1128/JVI.00974-10, PMID: 20631133 PMC2937655

[ref78] Spangle J. M. Munger K. (2013). The HPV 16 E6 oncoprotein causes prolonged receptor protein tyrosine kinase signalling and enhances internalisation of phosphorylated receptor species. PLoS Pathog. 9:e1003237. 10.1371/journal.ppat.1003237, PMID: 23516367 PMC3597533

[ref79] Stevanovic S. Draper L. M. Langhan M. M. Campbell T. E. Kwong M. L. Wunderlich J. R.. (2015). Complete regression of metastatic cervical cancer after treatment with human papillomavirus-targeted tumour-infiltrating T cells. J. Clin. Oncol. 33, 1543–1550. 10.1200/JCO.2014.58.9093, PMID: 25823737 PMC4417725

[ref80] Sun K. Salmon S. Yajjala V. K. Bauer C. Metzger D. W. (2014). Expression of suppressor of cytokine signalling 1 (SOCS 1) impairs viral clearance and exacerbates lung injury during influenza infection. PLoS Pathog. 10:e1004560. 10.1371/journal.ppat.1004560, PMID: 25500584 PMC4263766

[ref81] Tan S. Hougardy B. M. T. Meersma G. J. Schaap B. de Vries E. G. E. van der Zee A. G. J. (2012). HPV16 E6 RNA interference enhances cisplatin and death receptor –mediated apoptosis in human cervical carcinoma cells. Cancer J. Clin. 55, 74–108. 10.1124/mol.111.076539 22328720

[ref82] Thomas M. Banks L. (1999). Human papillomavirus (HPV) E6 interactions with Bak are conserved amongst E6 proteins from high and low risk HPV types. J. Gen. Virol. 80, 1513–1517. 10.1099/0022-1317-80-6-1513, PMID: 10374970

[ref83] Thomas J. T. Laimins L. A. (1998). Human papillomavirus oncoproteins E6 and E7 independently abrogate the mitotic spindle checkpoint. J. Virol. 72, 1131–1137. PMID: 9445009 10.1128/jvi.72.2.1131-1137.1998 PMC124587

[ref84] Thomas M. Laura R. Hepner K. Guccione E. Sawyers C. Lasky L.. (2002). Oncogenic human papillomavirus E6 proteins target the MAGI-2 and MAGI-3 proteins for degradation. Oncogene 21, 5088–5096. 10.1038/sj.onc.1205668, PMID: 12140759

[ref85] Tischer E. Mitchell R. Hartman T. Silva M. Gospodarowicz D. Fiddes J. C.. (1991). The human gene for vascular endothelial growth factor. Multiple protein forms are encoded through alternative exon splicing. J. Biol. Chem. 266, 11947–11954. PMID: 1711045

[ref86] Togtema M. Jackson R. Grochowski J. Villa P. L. Mellerup M. Chattopadhyaya J.. (2018). Synthetic siRNA targeting human papillomavirus 16 E6: a perspective on in vitro nanotherapeutic approaches. Nanomedicine 13, 455–474. 10.2217/nnm-2017-0242, PMID: 29382252

[ref87] Torre L. A. Islami F. Siegel R. L. Ward E. M. Jemal A. (2017). Global cancer in woman: burden and trends. Cancer Epidemiol. Biomarkers Prev. 26, 444–457. 10.1158/1055-9965.EPI-16-0858, PMID: 28223433

[ref88] Toussaint-Smith E. Donner D. B. Roman A. (2004). Expression of human papillomavirus type 16 E6 and E7 oncoproteins in primary foreskin keratinocytes is sufficient to alter the expression of angiogenic factors. Oncogene 23, 2988–2995. 10.1038/sj.onc.1207442 14968115

[ref89] Trimble C. L. Morrow M. P. Kraynyak K. A. Shen X. Dallas M. Yan J.. (2015). Safety, efficacy, and immunogenicity of VGX-3100, a therapeutic synthetic DNA vaccine targeting human papillomavirus 16 and 18 E6 and E7 proteins for cervical intraepithelial neoplasia 2/3: a randomised, double-blind, placebo-controlled phase 2b trial. Lancet 386, 2078–2088. 10.1016/S0140-6736(15)00239-1, PMID: 26386540 PMC4888059

[ref90] Varilla V. Atienza J. Dasanu C. A. (2013). Immune alterations and immunotherapy prospects in head and neck cancer. Expert. Opin. Biol. Ther. 13, 1241–1256. 10.1517/14712598.2013.810716 23789839

[ref91] Veldman T. Liu X. Yuan H. Schlegel R. (2003). Human papillomavirus E6 and Myc proteins associate in vivo and bind to and cooperatively activate the telomerase reverse transcriptase promoter. Proc. Natl. Acad. Sci. USA 100, 8211–8216. 10.1073/pnas.1435900100 12821782 PMC166208

[ref421] Venuti A. Paolini F. Nasir L. Corteggio A. Roperto S. Campo M. S.. (2011). Papillomavirus E5: The smallest oncoprotein with many functions. Mol. Cancer 10:140. 10.1186/1476-4598-10-140, PMID: 22078316 PMC3248866

[ref92] Wang N. Zhan T. Ke T. Huang X. Ke D. Campo M. S.. (2014). Increased expression of RRM2 by human papillomavirus E7 oncoprotein promotes angiogenesis in cervical cancer. Br. J. Cancer 110, 1034–1044. 10.1038/bjc.2013.817, PMID: 24423925 PMC3929894

[ref93] White E. A. Kramer R. E. Tan M. J. A. Hayes S. D. Harper J. W. Howley P. M. (2012a). Comprehensive analysis of host cellular interactions with human papillomavirus E6 proteins identifies new E6 binding partners and reflects viral diversity. J. Virol. 86, 13174–13186. 10.1128/JVI.02172-12 23015706 PMC3503137

[ref94] White E. A. Sowa M. E. Tan M. J. A. Jeudy S. Hayes S. D. Santha S. (2012b). Systematic identification of interactions between host cell proteins and E7 oncoproteins from diverse human papillomaviruses. Proc. Natl. Acad. Sci. USA 109, E260–E267. 10.1073/pnas.1116776109 22232672 PMC3277141

[ref95] Wright A. V. Nunez J. K. Doudna J. A. (2016). Biology and applications of CRISPR systems: harnessing nature’s toolbox for genome engineering. Cell 164, 29–44. 10.1016/j.cell.2015.12.035, PMID: 26771484

[ref96] Wyman C. Kanaar R. (2006). DNA double-strand break repair: all’s well that ends well. Annu. Rev. Genet. 40, 363–383. 10.1146/annurev.genet.40.110405.090451, PMID: 16895466

[ref98] Xiong Y. Kuppuswamy D. Li Y. Livanos E. M. Hixon M. White A.. (1996). Alteration of cell cycle kinase complexes in human papillomavirus E6- and E7-expressing fibroblasts precedes neoplastic transformation. J. Virol. 70, 999–1008. PMID: 8551641 10.1128/jvi.70.2.999-1008.1996 PMC189905

[ref99] Yamato K. Yamada T. Kizaki M. Ui-Tei K. Natori Y. Fujino M.. (2008). New highly potent and specific E6 and E7 siRNAs for treatment of HPV 16 positive cervical cancer. Cancer Gene Ther. 15, 140–153. 10.1038/sj.cgt.7701118, PMID: 18157144

[ref100] Yang A. Jeang J. Cheng K. Cheng T. Yang B. Wu T. C.. (2016). Current state in the development of candidate therapeutic HPV vaccines. Expert Rev. Vaccines 15, 989–1007. 10.1586/14760584.2016.1157477, PMID: 26901118 PMC4977850

[ref101] Yoshiba T. Saga Y. Urabe M. Uchibori R. Matsubara R. Fujiwara H. (2018). CRISPR/Cas-9 mediated cervical cancer treatment targeting human papillomavirus E6. Oncol. Lett. 17, 2197–2206. 10.3892/ol.2018.9815 30675284 PMC6341785

[ref102] Yuan C. H. Filippova M. Duerksen-Hughes P. (2012). Modulation of apoptotic pathways by human papillomaviruses (HPV): mechanisms and implications for therapy. Viruses 4, 3831–3850. 10.3390/v4123831, PMID: 23250450 PMC3528293

[ref103] Zanier K. ould M’hamed ould Sidi A. Boulade-Ladame C. Rybin V. Chappelle A. Atkinson A.. (2012). Solution structure analysis of the HPV16 E6 oncoprotein reveals a self-association mechanism required for E6-mediated degradation of p53. Structure 20, 604–617. 10.1016/j.str.2012.02.001, PMID: 22483108 PMC3325491

[ref104] Zhang B. Chen W. Roman A. (2006). The E7 proteins of low- and high-risk human papillomaviruses share the ability to target the pRB family member p130 for degradation. Proc. Natl. Acad. Sci. USA 103, 437–442. 10.1073/pnas.0510012103 16381817 PMC1326189

[ref105] Zhang Y. Dakic A. Chen R. Dai Y. Schlegel R. Liu X. (2017). Direct HPV E6/Myc interactions induce histone modifications, pol II phosphorylation, and hTERT promoter activation. Oncotarget 8, 96323–96339. 10.18632/oncotarget.22036, PMID: 29221209 PMC5707103

[ref106] Zheng Y. F. Rao Z. G. Zhang J. R. (2002). Effects of anti-HPV16 E6-ribozyme on the proliferation and apoptosis of human cervical cancer cell line CaSKi. Di Yi Jun Yi Da Xue Xue Bao 22, 496–498. PMID: 12297466

[ref107] Zheng Y. Zhang J. Rao Z. (2004). Ribozyme targeting HPV16 E6E7 transcripts in cervical cancer cells suppresses cell growth and sensitizes cells to chemotherapy and radiotherapy. Cancer Biol. Ther. 3, 1129–1134. discussion 1135-1136. 10.4161/cbt.3.11.1215 15467442

[ref108] Zhou X. Munger K. (2009). Expression of the human papillomavirus type 16 E7 oncoprotein induces an autophagy-related process and sensitizes normal human keratinocytes to cell death in response to growth factor deprivation. Virology 385, 192–197. 10.1016/j.virol.2008.12.003, PMID: 19135224 PMC2673705

[ref109] Zhou X. Spangle J. M. Munger K. (2009). Expression of a viral oncoprotein in normal human epithelial cells triggers an autophagy-related process: is autophagy an “Achilles’ heel” of human cancers? Autophagy 5, 578–579. 10.4161/auto.5.4.8367 19333004

